# A One Health perspective to identify environmental factors that affect Rift Valley fever transmission in Gezira state, Central Sudan

**DOI:** 10.1186/s41182-019-0178-1

**Published:** 2019-11-27

**Authors:** Rania Salah Eldien Bashir, Osama Ahmed Hassan

**Affiliations:** 1 Animal Health Directorate, General Directorate of Animal Health and Epizootic Diseases Control, Ministry of Livestock, Khartoum, Sudan; 2 The Centre for Global Health, Department of Community Medicine and Global Health, Faculty of Medicine, University of Oslo, Oslo, Norway; 3 Department of Clinical Microbiology, Unit of Virology, Faculty of Medicine, Umeå University, Umeå, Sweden

**Keywords:** Rift Valley fever, One Health, Remote sensing, Geographical information system, Normalized Difference Vegetation Index, Soil type, Multilevel logistic regression, Gezira state, Sudan

## Abstract

**Background:**

Rift Valley fever (RVF) is a zoonotic viral vector-borne disease that affects both animals and humans and leads to severe economic consequences. RVF outbreaks are triggered by a favorable environment and flooding, which enable mosquitoes to proliferate and spread the virus further. RVF is endemic to Africa and has spread to Saudi Arabia and Yemen. There is great concern that RVF may spread to previously unaffected geographic regions due to climate change. We aimed to better understand the spatiotemporal pattern of the 2007 RVF outbreak at the human–animal–environment interface and to determine environmental factors that may have effects on RVF occurrence in Gezira state, Sudan.

**Materials and methods:**

We compiled epidemiological, environmental, and spatiotemporal data across time and space using remote sensing and a geographical information system (GIS). The epidemiological data included 430 RVF human cases as well as human and animal population demographic data for each locality. The cases were collected from 41 locations in Gezira state. The environmental data represent classified land cover during 2007, the year of the RVF outbreak, and the average of the Normalized Difference Vegetation Index (NDVI) for 6 months of 2007 is compared with those of 2010 and 2014, when there was no RVF outbreak. To determine the effect of the environmental factors such as NDVI, soil type, and RVF case’s location on the Blue Nile riverbank on RVF incidence in Gezira state, a multilevel logistic regression model was carried out.

**Results:**

We found that the outbreak in Gezira state occurred as a result of interaction among animals, humans, and the environment. The multilevel logistic regression model (*F* = 43,858, df = 3, *p* = 0.000) explained 23% of the variance in RVF incidence due to the explanatory variables. Notably, soil type (*β* = 0.613, *t* = 11.284, *p* = 0.000) and NDVI (*β* = − 0.165, *t* = − 3.254, *p* = 0.001) were the explanatory environmental factors that had significant effects on RVF incidence in 2007 in Gezira state, Sudan.

**Conclusions:**

Precise remote sensing and the GIS technique, which rely on environmental indices such as NDVI and soil type that are satellite-derived, can contribute to establishing an early warning system for RVF in Sudan.

Future preparedness and strengthening the capacity of regional laboratories are necessary for early notification of outbreaks in animals and humans.

## Introduction

Rift Valley fever (RVF) is a zoonotic viral vector-borne disease that primarily affects animals [1, 2]. The disease is transmitted to humans through direct contact with an infected animal or its products [3]. In addition, bites by mosquitoes of the *Aedes* and *Culex* genera transmit the disease among animals and between animals and humans [4–8].

RVF causes serious health and economic problems. In animals, RVF affects different species and causes death particularly in lambs [9], and in pregnant livestock, RVF can cause abortion [9, 10]. This often leads to severe socioeconomic impacts in affected countries [11–13]. In humans, RVF varies from mild to severe symptoms such as renal failure, encephalitis, vison problems, hemorrhage, and death [14–17]. RVF infection also reflects a maternal health concern, as it has been found to be associated with miscarriage in pregnant women [18]. Economically, RVF outbreaks have resulted in jeopardization of the livestock trade and food insecurity [19, 20].

Since Rift Valley fever virus (RVFV) was first discovered in Kenya in 1930 [1], the virus has adapted to different ecological zones [21]. Accordingly, RVF has expanded geographically both in and outside of Africa [22]. This explains the raised concern that RVF could spread to new unaffected regions by transport of livestock, human travel, land-use changes, and/or climate change [23–25].

During 2006–2008, a wave of RVF outbreaks occurred in central and east Africa, which included Sudan, Kenya, Somalia, Tanzania, and Madagascar. The outbreaks resulted in an estimated 230,000 human cases [26]. The 2006–2008 RVF outbreaks were predicted at the regional level of east Africa using a remote sensing model. However, the prediction had low accuracy in Sudan [20, 26]. We hypothesize that understanding local environmental conditions during RVF outbreaks in Sudan could improve outbreak predictions in the future. Many scientific findings have proven that studying the spatiotemporal patterns of infectious diseases is useful to understand the diseases’ geographical distributions and possible control strategies [27–33]. In line with this, our study, with the aid of a geographical information system (GIS) and remote sensing (RS), aimed to better understand the spatial and temporal patterns of the 2007 RVF outbreak and to examine the effect of environmental factors on RVF incidence on the country subscale level in Gezira state, Sudan.

## Materials and methods

### Study area

**Fig. 1:**
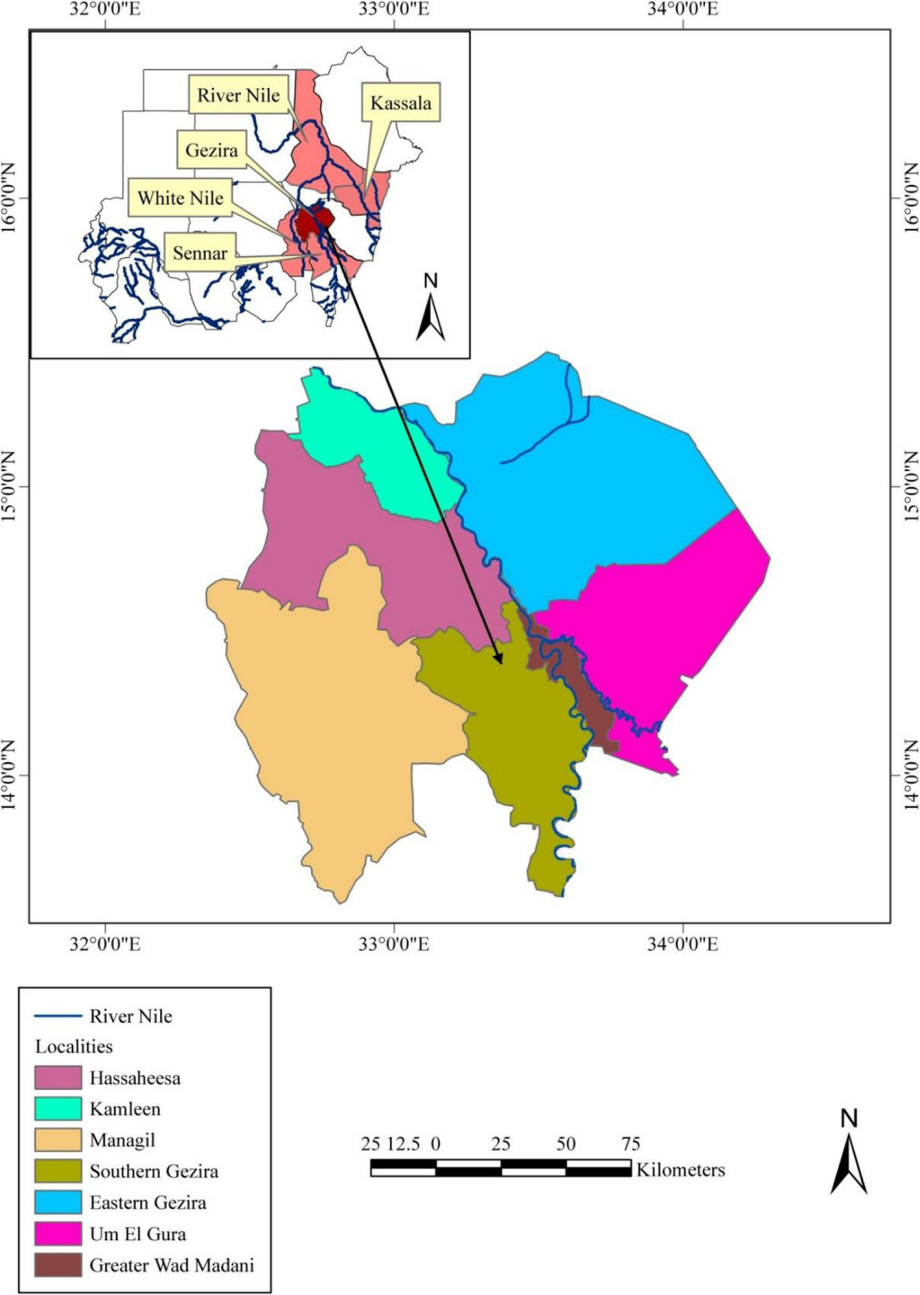
Map of Sudan showing Gezira state and its localities. The data frame displays other states affected by the 2007 RVF outbreak

Gezira is one of the 18 states of Sudan. The state lies in the central part of the country between the Blue Nile and the White Nile. The state’s area is about 27,549 km^2^, with a population of 3,734,320 according to the 2008 census. Gezira is home to one of the biggest agricultural schemes in Africa and the Middle East, which began to foster cotton farming in 1925. Gezira is divided administratively into seven localities and shares a border with Khartoum (the capital of Sudan), as well as three other agricultural states (Fig. 1).

### Approach, datasets, and process

**Fig. 2:**
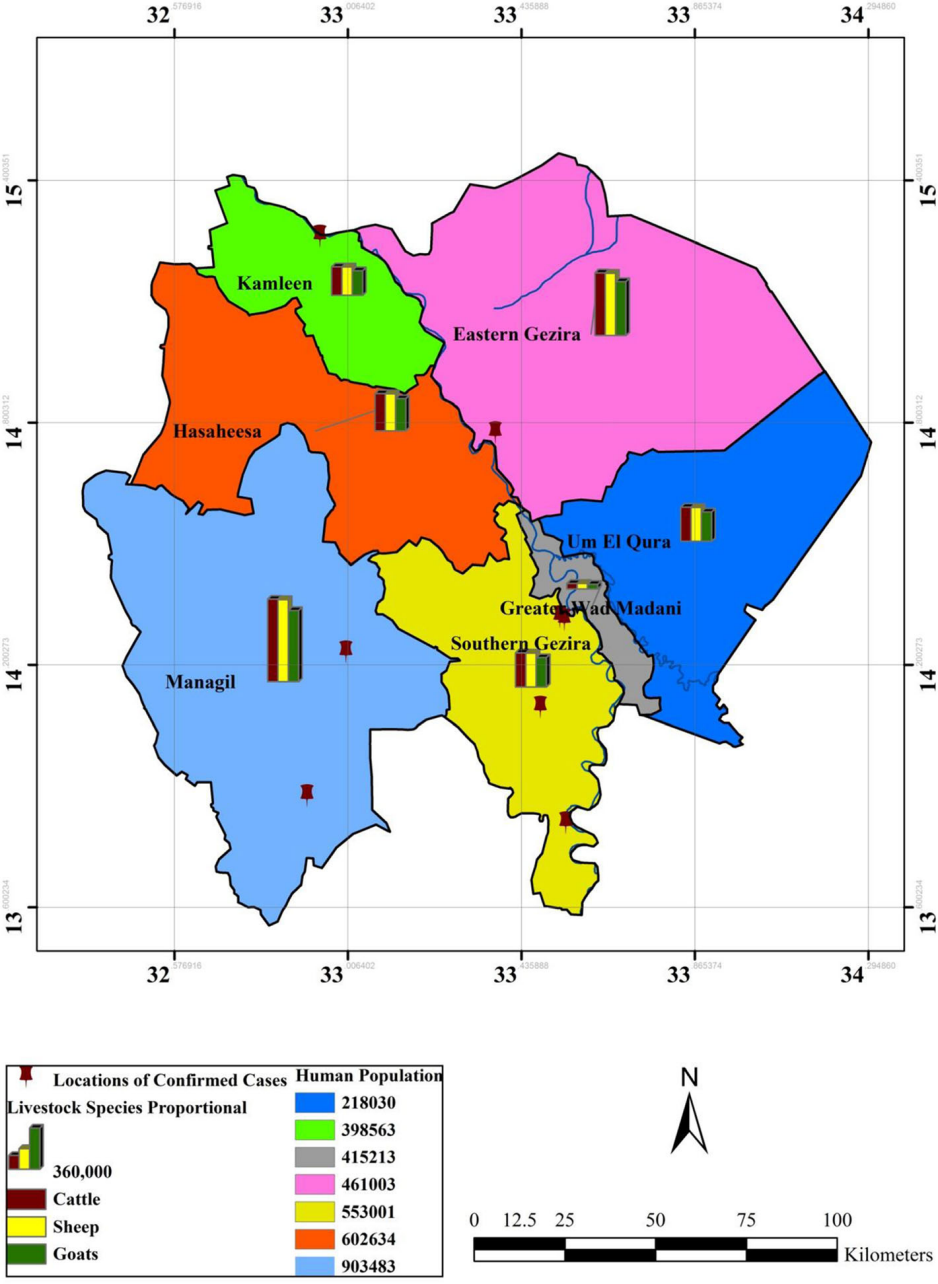
Spatial distribution of RVF human confirmed cases among Gezira state localities and the related animal population as a proportional bar column chart and human populations

**Fig. 3:**
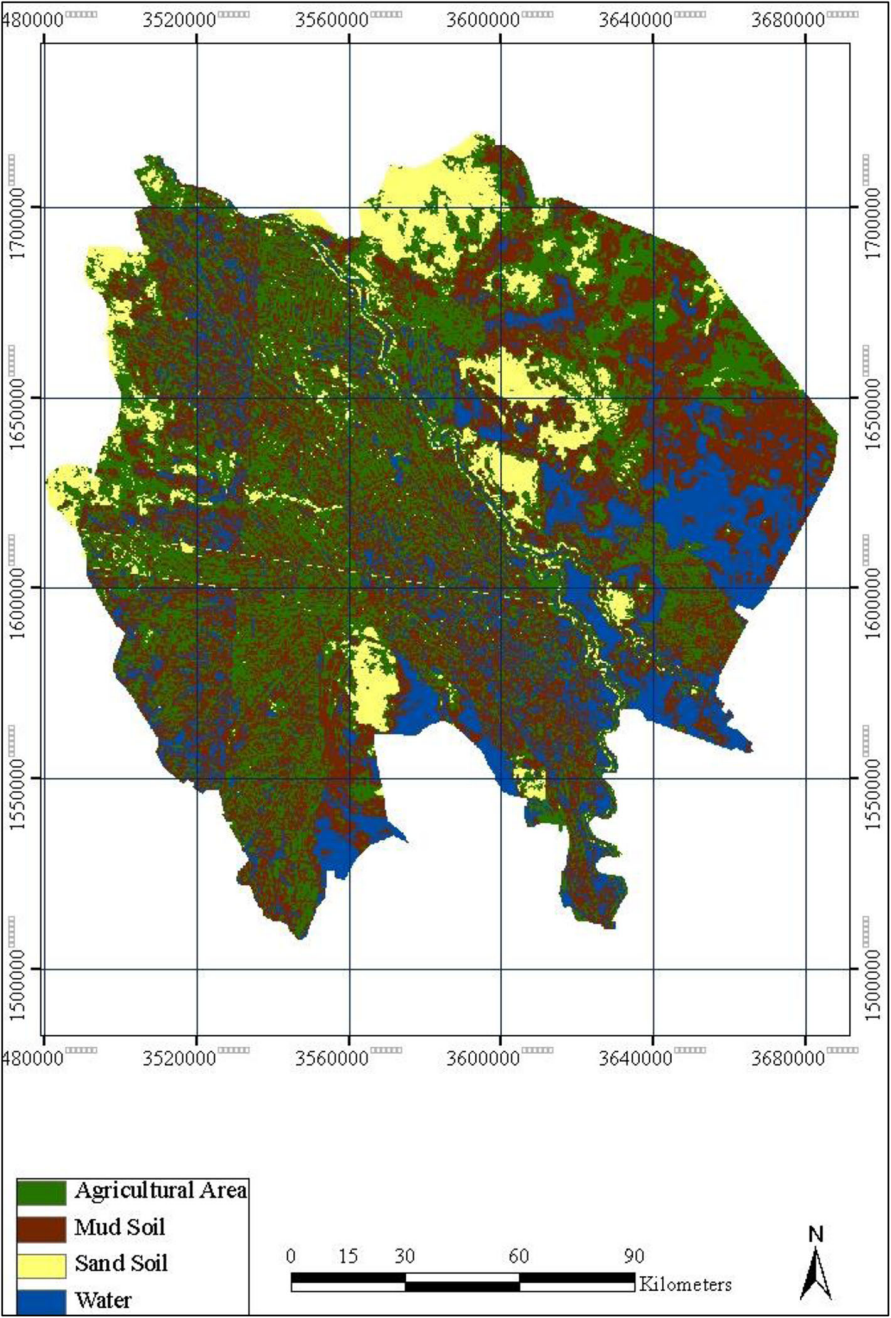
Gezira land cover classification in December 2007

RVF occurred at the human–animal–environment intersection, and we collected data regarding these three pillars (Figs. 2, 3, and 5). Such an integrated approach is called “One Health,” which emphasizes the connection of animal, human, and environmental health [34]. The study used epidemiological and spatiotemporal data of RVF cases’ occurrence as well as environmental data.

Epidemiological data such as RVF cases in humans and the human population (the number of individuals per square mile) were collected from the Gezira state Ministry of Health. Similarly, the animal population data were collected from the federal Ministry of Livestock. We collected the data as figures by locality, and we show the animal population in the maps as a bar chart (proportional) and the human population as a color gradient to differentiate between the localities (Fig. 2). All data were distributed by locality, combined, and incorporated into GIS. This was done to create a map from the three layers of the locations of human cases and the human and animal populations of each locality in Gezira state.

RS usually enables one to obtain data without direct contact with the ground and transfer the data to an image via satellite sensors [35, 36]. RS data include land cover, such as water, soil type, and agricultural areas, obtained by the Moderate Resolution Imaging Spectroradiometer (MODIS) at the National Aeronautics and Space Administration (NASA) (http://reverb.echo.nasa.gov/reverb/). The RS data was used to produce land cover classifications for Gezira state in the Earth Resources Data Analysis System (ERDAS) 8.5 with raster calculation tools and supervised classification. The Normalized Difference Vegetation Index (NDVI) is a vegetation index that is collected on a daily basis by MODIS satellite image at a resolution of 250 m and is available on the NASA website (http://reverb.echo.nasa.gov/reverb/). We used ArcGIS 9.3 to produce an image with three bands. In addition, we used ERDAS 8.5 to produce an NDVI image. The value of NDVI was obtained by the eq. NDVI = (near IR − red band)/(near IF+ red band), MODIS image: band 1, 2. Band 1(0.62–0.67), band 2(0.841–0.876). We calculated the mean of NDVI for each month from the low and high values for 6 months for each year of 2007, 2010, and 2014 for Gezira state. The months included August and September for autumn, April and May for summer, and November and January for winter. In addition, NDVI of 2007, when the outbreak occurred, was compared with those of 2010 and 2014 in Gezira. We selected 2010 and 2014 as control comparative years due to the absence of RVF outbreaks as well as the availability of data to calculate NDVI for Gezira state. To examine the effect of environmental factors on the occurrence of RVF, a multilevel linear regression model using SPSS version 25 was carried out. The model used RVF incidence as a dependent (outcome) variable while NDVI, soil type, and RVF case’s location as independent (explanatory) variables. RVF incidence was classified as suspected, probable, or confirmed (suspected, 0; probable, 1; confirmed, 2). The explanatory variables were classified as sand versus mud soil (sand, 0; mud, 1) and east versus west bank of the Blue Nile river for location (east, 0; west, 1). We calculated NDVI for each locality, then calculated the average, and we considered values < 0.3 as low, while those > 0.3 were high (in the model, low 0; high 1).

Since GIS and RS are tools that help to connect epidemiological data with climatic and spatial data [35, 36], the epidemiological and RS datasets were imported into ArcGIS 9.3 as either raster or shape-file formats. The datasets were re-projected to the WGS 84 datum surface and clipped to an area extending to latitudes 13–15° N and longitudes 34–32.5° E, corresponding to the geographical limits of Gezira.

## Results

During the 2007 RVF outbreak, between October and November, the Ministry of Health in Gezira reported 430 human cases from 41 locations. In this study, we classified the cases as confirmed, probable, and suspected. A confirmed case was confirmed by a laboratory test. A probable case met the clinical case definition of RVF but was not confirmed by a laboratory test. A suspected case showed a similar clinical case definition, but its confirmatory test had a negative result (Additional file 1).

Gezira is divided by the Blue Nile River, and the reported cases spread across both the eastern and western sides. However, the majority of the confirmed and probable cases were registered on the western side (Additional file 1). The western side also maintained higher animal and human populations compared with the eastern side. This was the case for the Managil locality, which experienced the highest number of human probable cases. The Managil locality is situated on the western side and possesses the highest animal and human populations within Gezira state (Additional file 1, Fig. 2). In contrast, other localities on the western side encountered fewer confirmed and probable cases of RVF, but they maintained smaller animal and human populations (Fig. 2 and Additional file 1).

The land cover satellite image of Gezira state during 2007 revealed that the western localities were very green compared with the eastern localities (Fig. 3). The prevalent soil was mud in the west and sand in the east (Fig. 3). In addition to the Blue Nile, which flows through the state, there was a lot of surface water in both the eastern and western parts of Gezira state, as is shown by the satellite image (Fig. 3).

**Fig. 4:**
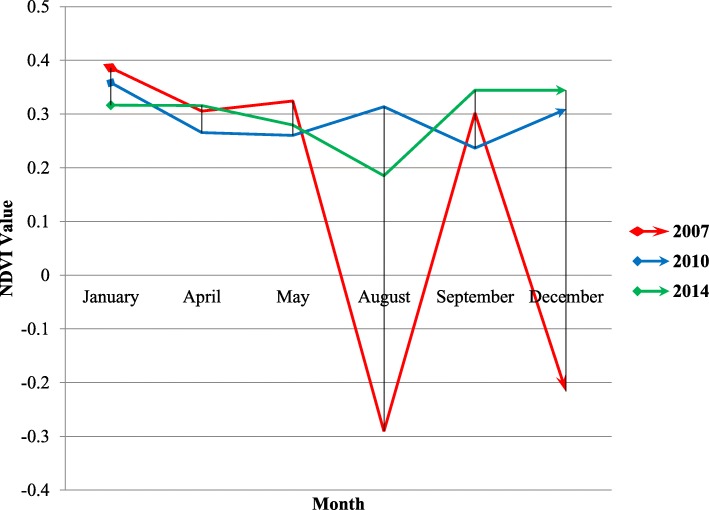
Average NDVI for the year 2007 compared with 2010 and 2014

In Gezira in 2007, NDVI changed significantly from a negative value (− 0.3) in August to a positive value (0.3) in September. This was similar to the change from − 0.2 in December to 0.4 in January 2007. In contrast, NDVI only changed slightly in the same period in 2010 and 2014 (Fig. 4 and Additional files 2 and 3).

**Fig. 5:**
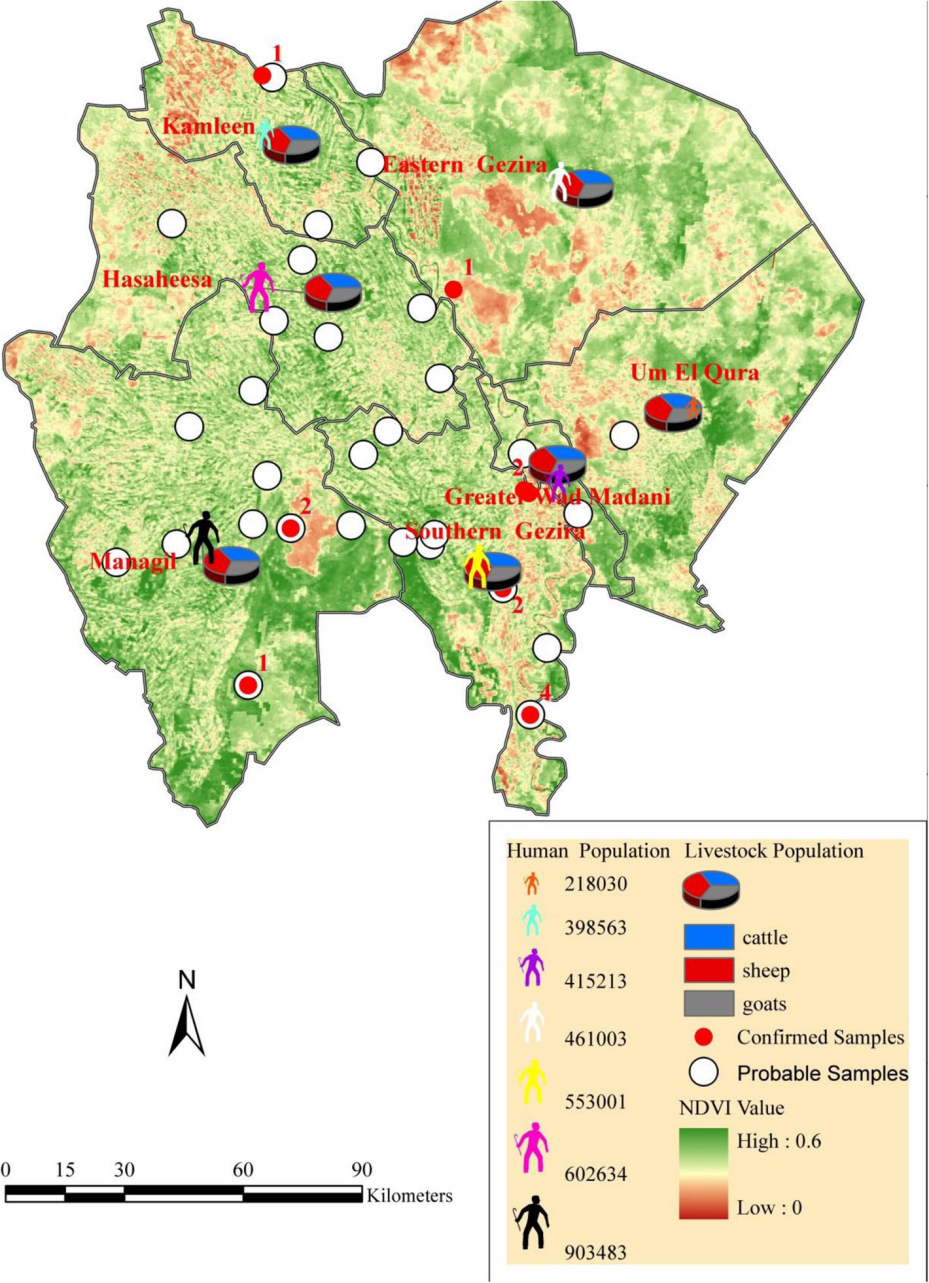
NDVI for Gezira state overlaid with RVF human cases (confirmed, probable) and the animal population as a pie chart (percentage) and human populations as a simple gradient

NDVI was also analyzed together with the pattern of human RVF cases and the human and livestock populations in Gezira state. In Gezira, the livestock consisted of cattle, sheep, and goats, with the latter species the most populous (Fig. 5).

The NDVI was higher in localities that encountered a higher number of RVF human cases and possessed larger human and animal populations, for example, in Managil and Southern Gezira (Fig. 5).

In order to ascertain the extent to which NDVI, soil types, and RVF cases’ locations can explain RVF transmission in Gezira state, a multilevel linear regression was carried out. After checking the assumptions of the logistic regression, the model determined that 23% of the variation in RVF incidence was explained by the explanatory variables (*R*^2^ = 0.236). The model was significant and explained RVF incidence (*F* = 43,858, df = 3, *p* = 0.000). The model revealed that mud soil was the strongest explanatory variable, as it had a significant effect on RVF incidence (*β* = 0.613, *t* = 11.284, *p* = 0.000; Table 1, Additional file 4). On the other hand, a significant negative effect of NDVI was found, showing that RVF incidence was less affected by NDVI in comparison with soil type (*β* = − 0.165, *t* = − 3.254, *p* = 0.001; Table 1, Additional file 4). In contrast, RVF case’s location had no significant effect on RVF incidence (*β* = − 0.011, *t* = − 0.402, *p* = 0.688; Table 1, Additional file 4).

**Table 1:** Summary of regression model coefficient

	*β*	Std. error	*T*	Sig.
Constant	0.589	0.051	11.622	0.000*
NDVI (low versus high)	− 0.165	0.051	− 3.254	0.001*
Soil type (sand versus mud)	0.613	0.054	11.284	0.000*
RVF case’s location (east versus west)	− 0.011	0.027	− 0.402	0.688

*Significant value (*p* < 0.05)

Dependent variable: RVF incidence

## Discussion

The 2007 RVF outbreak spread in all the localities of Gezira state, Sudan. The Managil locality, where the highest number of probable cases was found, shares its border with White Nile state. In fact, the first human index case of the 2007 RVF outbreak in Sudan was detected in White Nile state [26]. One could suspect that the virus was transmitted from White Nile state to the Managil locality in Gezira state, through, e.g., livestock movement and/or infected mosquitoes. Animal movement has been connected to the spread of RVFV to new areas in other countries [37]. Likewise, the Southern Gezira and Hasaheesa localities are proximate to the Managil locality; such proximity may facilitate the transmission of RVFV and explain why the highest number of probable human RVF cases existed there. Generally, most of the affected areas were located on the west bank of the Blue Nile. Although the Blue Nile may act as natural barrier against RVFV, location was not found to be an explanatory variable for the 2007 RVF incidence in the multilevel regression model (*p* = 0.688) (Table 1, Additional file 4).

Most confirmed human cases were detected in the Southern Gezira locality. Southern Gezira is located near the capital of Gezira state, Wad Madani, and has access to RVFV diagnostic tests. This could explain why Southern Gezira had more confirmed human RVF cases than other localities. On the contrary, the Managil locality had the highest number of probable cases. The lack of a confirmatory diagnostic test for the probable cases in Managil could be due to the lack of infrastructure and laboratory expertise at Managil’s rural hospital. In addition, Managil is located in a remote part of Gezira. Therefore, the option to send the samples to the capital of Gezira to analyze them for RVFV was difficult due to rough roads during the rainy season. The challenge to confirm probable RVF cases has been seen for other outbreaks during heavy rains, such as the 2008 RVF outbreak in Madagascar [38]. Better access to proper diagnostic capacity is needed to confront future RVF outbreaks.

In 2007, a survey was carried out in Gezira to identify the seroprevalence of RVFV in livestock. The result indicated that the overall prevalence of RVFV was 50% in livestock in Gezira. Out of the 220 livestock sampled and tested for Immunoglobulin M (IgM), there were 103 goats, 74 sheep, and 43 cattle. The specific prevalence in goats, sheep, and cattle was 61%, 51%, and 23%, respectively [39]. These results indicate that livestock could be involved in the amplification of RVFV in Gezira. The most infected species was goats, while in other RVF outbreaks, such as in Uganda, cattle played a more important role [10]. Unfortunately, the result of the survey was not distributed at the locality level of Gezira state in order to incorporate it in Fig. 2 and Additional file 1.

Regarding the land cover analysis of Gezira state, the remote sensing satellites revealed that most of the localities that suffered from RVF had features that suggested agriculture activities. For instance, agricultural production was practiced on mud soil, while less affected localities were covered by sandy soil, with fewer agricultural activities. Interestingly, mud soil was found to be a strong explanatory factor for RVF incidence in Gezira state, Sudan (*p* = 0.000) (Table 1, Additional file 3). Mud soil is known to have low penetration capacity, so it maintains water at its surface. This could enhance the breeding of RVFV mosquito vectors due to the formation of dambos, as has been shown for other RVF outbreaks in Kenya [40, 41].

Concerning NDVI, it normally ranges from + 1 to − 1. Areas of barren rock and sand usually show very low NDVI values (for example, 0.1 or less). Sparse vegetation such as shrubs and grasslands or senescing crops may result in moderate NDVI values (approximately 0.2 to 0.5). High NDVI values (approximately 0.6 to 0.9) correspond to dense vegetation, such as that found in temperate and tropical forests or crops at their peak growth stage [26]. When we examined the pattern of the 2007 NDVI, it changed dramatically from very dry in May to extremely wet in August, back to dry in September, and then again to very wet in December 2007. In 2010 and 2014, NDVI did not change similarly but was instead stable in a dry phase. The dramatic change of NDVI during autumn 2007 may explain why there were better conditions for RVF to occur in 2007 and not in 2010 and 2014 in Gezira state. The heavy rains in August and December resulted in extremely wet mud soil in Gezira, a state with high populations of both livestock and humans, leading to extreme flooding and favorable conditions for RVFV mosquito vectors.

Our result shows that the significant variance in NDVI during the autumn period may point to an increased risk of RVF outbreaks. We recommend that further studies be conducted in other countries to examine such associations. In agreement with this, NDVI was found to be a second environmental factor that influenced the 2007 RVF incidence in Gezira state (*p* = 0.001) (Table 1). This result is similar to a study that was conducted in Kenya, where NDVI was found to be an indicative index to predict RVF outbreaks between 1982 and 1997 [42]. Similarly, NDVI succeeded in predicting RVF outbreak events between 1950 and 1998 in another a retrospective study in Kenya when used with Pacific and Indian Ocean sea surface temperature (SST) index anomalies [43]. In contrast, NDVI was less associated with RVF outbreak occurrence in South Africa between 2008 and 2011 [44].

Both soil type and NDVI could be good indicators to include in an early warning system for RVF outbreaks in Sudan.

This study has some limitations. The study used livestock populations as a factor related to the amplification of RVFV. However, details about the numbers and locations of animals affected during the 2007 RVF outbreak in Gezira state are lacking. If such data were available, they would give us a better understanding of the outbreak dynamics. Similarly, we lack data about the mosquito species that prevailed in Gezira state during the outbreak. Both animal cases and the type of mosquitoes that were involved would help to better explain the RVF outbreak pattern at the human–animal–environment interface. We hope that such data will be available in the future. Moreover, the small sample size (430 cases) may have affected the fitness of our multilevel logistic regression model (*R*^2^ = 0.236). A bigger sample size could have increased the goodness of fit for the model. However, the 2007 RVF outbreak was one of the biggest outbreaks in the country and the region, and it would be difficult to get a bigger sample size. Therefore, the model is reasonable within such context.

## Conclusions

The primary goal of this study was to better understand the spatiotemporal patterns of an RVF outbreak and to examine the possible effects of environmental factors such as NDVI, soil type, and RVF case’s location on RVF occurrence on the subscale country level in Gezira state, Sudan. The collection of data at the human–animal–environmental interface assisted our understanding of RVF from the One Health perspective.

The combination of epidemiological, spatiotemporal, and environmental data such as land cover and NDVI using remote sensing and geographical information systems provided insights into the RVF incidences in the local conditions of Gezira. Notably, mud soil and higher level of NDVI as environmental parameters can indicate increased risk of RVF. Principally, there was inductive interaction among animals, humans, and the conducive environment, and that could explain the occurrence of the 2007 RVF outbreak in Gezira.

Getting access to and strengthening regional laboratories are crucial for case notification of RVF in both animals and humans. Without these steps, the timely control of future RVF outbreaks will be difficult, even if they are predicted.

## Supplementary information

**Additional file 1: Table S1.** The 2007 RVF outbreak human cases distributed by localities in Gezira state, Sudan.

**Additional file 2: Table S2.** Type, source, and resolution of study dataset.

**Additional file 3: Table S3.** Average NDVI during 2007, 2010, and 2014 in Gezira state, Sudan.

**Additional file 4: Table S4.** Detailed Multilevel Linear Regression Model. Model Summary^b^.

## Data Availability

The datasets used and/or analyzed in the current study are available from the corresponding author upon reasonable request.

## Abbreviations

ERDAS: Earth resources data analysis system
GIS: Geographical information system
IgM: Immunoglobulin M
MODIS: Moderate resolution imaging spectroradiometer
NDVI: Normalized Difference Vegetation Index
RS: Remote sensing
RVF: Rift Valley fever
RVFV: Rift Valley fever virus
SST: Indian Ocean sea surface temperature
WGS: World geodetic system
