# Supplementary material for: A One Health perspective to identify environmental factors that affect Rift Valley fever transmission in Gezira state, Central Sudan
Source: Trop Med Health. 2019 Nov 27;47:54. doi: 10.1186/s41182-019-0178-1 (PMC6880409; doi:10.1186/s41182-019-0178-1)
Supplement: Supplementary file 1 — Additional file 1: Table S1. The 2007 RVF outbreak human cases distributed by localities in Gezira state, Sudan. [file 41182_2019_178_MOESM1_ESM.docx]

**Additional file 1. The 2007 RVF outbreak human cases distributed by localities in Gezira state, Sudan**

| Locality | Location on the Blue Nile riverbank | Confirmed | Probable | Suspected |
| --- | --- | --- | --- | --- |
| Managil | West | 5 | 162 | 3 |
| Southern Gezira | West | 8 | 83 | 5 |
| Hasaheesa | West | 3 | 74 | 2 |
| Greater Wad Madani | West | 1 | 34 | 2 |
| Kamleen | West | 1 | 29 | 5 |
| Um El Qura | East | 0 | 9 | 1 |
| Eastern Gezira | East | 1 | 2 | 0 |
