# Supplementary material for: A One Health perspective to identify environmental factors that affect Rift Valley fever transmission in Gezira state, Central Sudan
Source: Trop Med Health. 2019 Nov 27;47:54. doi: 10.1186/s41182-019-0178-1 (PMC6880409; doi:10.1186/s41182-019-0178-1)
Supplement: Supplementary file 2 — Additional file 2: Table S2. Type, source, and resolution of study dataset. [file 41182_2019_178_MOESM2_ESM.docx]

**Additional file 2. Type, source, and resolution of study dataset**

| No | Type | Source | Spatiotemporal resolution |
| --- | --- | --- | --- |
| 1 | RVF human cases | Ministry of Health, Gezira state | Square mile |
| 2 | Animal population | Ministry of Agriculture and Livestock, Gezira state | Per locality |
| 3 | Human population | Federal Ministry of Health | Per locality |
| 5 | Land use | NASA (<http://reverb.echo.nasa.gov/reverb/>) | 250 meters |
| 6 | NDVI | MODIS | 250 meters |
