# Supplementary material for: A One Health perspective to identify environmental factors that affect Rift Valley fever transmission in Gezira state, Central Sudan
Source: Trop Med Health. 2019 Nov 27;47:54. doi: 10.1186/s41182-019-0178-1 (PMC6880409; doi:10.1186/s41182-019-0178-1)
Supplement: Supplementary file 3 — Additional file 3: Table S3. Average NDVI during 2007, 2010, and 2014 in Gezira state, Sudan. [file 41182_2019_178_MOESM3_ESM.docx]

**Additional file 3. Average NDVI during 2007, 2010, and 2014 in Gezira state, Sudan.**

| **Year** | **January** | **April** | **May** | **August** | **September** | **December** |
| --- | --- | --- | --- | --- | --- | --- |
| 2007 | 0.4 | 0.3 | 0.3 | -0.3 | 0.3 | -0.2 |
| 2010 | 0.4 | 0.3 | 0.3 | 0.3 | 0.2 | 0.3 |
| 2014 | 0.3 | 0.3 | 0.3 | 0.2 | 0.3 | 0.3 |
