# Supplementary material for: A One Health perspective to identify environmental factors that affect Rift Valley fever transmission in Gezira state, Central Sudan
Source: Trop Med Health. 2019 Nov 27;47:54. doi: 10.1186/s41182-019-0178-1 (PMC6880409; doi:10.1186/s41182-019-0178-1)
Supplement: Supplementary file 4 — Additional file 4: Table S4. Detailed Multilevel Linear Regression Model. Model Summaryb. [file 41182_2019_178_MOESM4_ESM.docx]

**Additional file 4. Detailed Multilevel Linear Regression Model**

**Model Summary^b^**

| Model | R | R^2^ | Adjusted R^2^ | Std. Error of the Estimate |
| --- | --- | --- | --- | --- |
| 1 | 0.486^a^ | 0.236 | 0.231 | 0.25759 |

| **ANOVA^a^** | | | | | | |
| --- | --- | --- | --- | --- | --- | --- |
| Model | | Sum of Squares | df | Mean Square | F | Sig. |
| 1 | Regression | 8.730 | 3 | 2.910 | 43.858 | 0.000^b^ |
|  | Residual | 28.267 | 426 | 0.066 |  |  |
|  | Total | 36.998 | 429 |  |  |  |
| a. Dependent Variable: RVF cases | | | | | | |
| b. Predictors: (Constant), location on the Blue Nile riverbank, Soil Type, NDVI | | | | | | |

| **Coefficients^a^** | | | | | | | | | | | |
| --- | --- | --- | --- | --- | --- | --- | --- | --- | --- | --- | --- |
| Model | | Unstandardized Coefficients | | Standardized Coefficients | t | Sig. | 95.0% Confidence Interval for B | | Collinearity Statistics | | |
|  |  | B | Std. Error | Beta |  |  | Lower Bound | Upper Bound | Tolerance | VIF |  |
| 1 | (Constant) | 0.589 | 0.051 |  | 11.622 | 0.000* | 0.490 | 0.689 |  |  |  |
|  | NDVI | -0.165 | 0.051 | -0.163 | -3.254 | 0.001* | -0.264 | -0.065 | 0.716 | 1.397 |  |
|  | Soil Type | 0.613 | 0.054 | 0.541 | 11.284 | 0.000* | 0.506 | 0.720 | 0.781 | 1.280 |  |
|  | Location on the Blue Nile riverbank | -0.011 | 0.027 | -0.018 | -0.402 | 0.688 | -0.064 | 0.042 | 0.898 | 1.113 |  |
| a. Dependent Variable: RVF cases  *Significant value (*p* < 0.05) | | | | | | | | | | | |
